# Conquer fear: protocol of a randomised controlled trial of a psychological intervention to reduce fear of cancer recurrence

**DOI:** 10.1186/1471-2407-13-201

**Published:** 2013-04-23

**Authors:** Phyllis N Butow, Melanie L Bell, Allan B Smith, Joanna E Fardell, Belinda Thewes, Jane Turner, Jemma Gilchrist, Jane Beith, Afaf Girgis, Louise Sharpe, Sophy Shih, Cathrine Mihalopoulos

**Affiliations:** 1 Psycho-Oncology Co-operative Research Group (PoCoG), School of Psychology, The University of Sydney, Sydney, NSW, 2006, Australia; 2 School of Medicine, University of Queensland, Herston, QLD, 4029, Australia; 3 Crown Princess Mary Cancer Centre, Westmead, NSW, 2145, Australia; 4 Royal Prince Alfred Hospital, Camperdown, NSW, 2050, Australia; 5 Ingham Institute for Applied Medical Research, South Western Sydney Clinical School, University of New South Wales, Liverpool, NSW, 2170, Australia; 6 School of Psychology, The University of Sydney, Sydney, NSW, 2006, Australia; 7 Health Economics Unit, Deakin University, Burwood, VIC, 3125, Australia

**Keywords:** Fear of cancer recurrence, Cancer, Oncology, RCT, S-REF model, Intervention, Metacognition, Detached mindfulness

## Abstract

**Background:**

Up to 70% of cancer survivors report clinically significant levels of fear of cancer recurrence (FCR). Despite the known negative impact of FCR on psychological wellbeing and quality of life, little research has investigated interventions for high FCR. Our team has developed and piloted a novel intervention (Conquer Fear) based on the Self-Regulatory Executive Function Model and Relational Frame Theory and is evaluating Conquer Fear in a randomised controlled trial (RCT). We aim to compare the efficacy and cost-efficacy of the Conquer Fear Intervention and relaxation training in reducing the impact of FCR.

**Methods/design:**

This study is a multi-centre RCT with 260 participants randomised either to the Conquer Fear Intervention or relaxation training. Both interventions will be delivered in five sessions over 10 weeks by trained psychologists, psychiatrists and social workers with five or more years experience in oncology. Conquer Fear sessions use attentional training, detached mindfulness, meta-cognitive therapy, values clarification and psycho-education to help patients change the way they regulate and respond to thoughts about cancer recurrence. Relaxation training includes training in progressive and passive muscle relaxation, meditative relaxation, visualisation and “quick relaxation” techniques. Relaxation was chosen to control for therapist time and attention and has good face-validity as an intervention. The primary outcome is fear of cancer recurrence. Secondary outcomes include distress, quality of life, unmet needs, and health care utilisation. Participants complete questionnaires prior to starting the intervention, immediately after completing the intervention, 3 and 6 months later. Eligible participants are early-stage breast or colorectal cancer survivors who have completed hospital-based treatment between 2 months and 5 years prior to study entry and report a score in the clinical range on the Fear of Cancer Recurrence Inventory. The biostatistician is blinded to group allocation and participants are blinded to which intervention is being evaluated. Randomisation is computer generated, stratified by therapist, and uses sequentially numbered sealed envelopes.

**Discussion:**

If successful, the study will provide an evidence-based intervention to reduce psychological morbidity in cancer survivors, and reduce overall health care costs due to more appropriate use of follow-up care and other health services in this very large population.

**Trial registration:**

Trial registration: ACTRN12612000404820

## Background

Improved medical treatments have led to higher survival rates for many cancers. According to recent estimates, about 2% of the Australian population is currently living with a diagnosis of a malignant cancer [1]. In 2004 there were approximately 655,000 Australian cancer survivors diagnosed with cancer during the previous 23 years [2]. In 2005, the US Institute of Medicine released a landmark report *From Cancer Patient to Cancer Survivor: Lost in Transition*[3]. The report was critical of the paucity of intervention research to address the psychosocial consequences of cancer and its treatments, and stated that “addressing survivors’ unmet needs and providing greater clarity around follow-up is likely to lead to significant efficiencies in health care delivery and potential cost savings.” [3]. One of the most prevalent unmet needs in survivors is for help with fear of cancer recurrence (FCR), defined as the fear that cancer could return or progress in the same place or another part of the body [4]. Several large studies have found that 21-40% of cancer survivors report a need for help dealing with FCR [5-7].

### Prevalence and trajectory of FCR

High levels of FCR are very common, with 42-70% of survivors reporting clinically significant levels of FCR [8,9]. Of 1442 adult cancer survivors recruited through Australian cancer registries, 46% worried about the cancer returning or getting worse at 12 months post-diagnosis [10]. Longitudinal studies of cancer survivors show that FCR usually does not decrease over time [11-15] even when risk of recurrence is low. This finding is supported by two reviews including both cross-sectional and longitudinal studies, which found little evidence for a relationship between time since diagnosis and FCR [16,17]. The enduring nature of FCR is exemplified by a study of long-term testicular cancer survivors (average time since diagnosis 11 years), which found that despite this population having a relatively low risk of relapse, 24% reported FCR bothered them ‘quite a bit’ and 7% that it bothered them ‘very much’ [18].

### The importance of evidence-based treatments for FCR

FCR has a functional impact on the lives of cancer survivors and their families. FCR is associated with poorer quality of life and emotional wellbeing; higher mental and physical fatigue; and higher depression and anxiety in cancer survivors [17]. In a recently published study of 218 younger patients with a history of early breast cancer, of the 70% reporting clinical levels of FCR, 25% said FCR considerably impaired their mood and 19% reported it considerably impaired their ability to make plans and set future goals [9]. Some studies have reported higher levels of FCR in caregivers than patients [19-21].

A survey of 76 psycho-oncology health professionals and 47 oncologists and nurses found that 30% reported FCR was an issue for more than half their patients; 31% of doctors reported spending more than 25% of the time in follow-up consultations discussing FCR; 46% found dealing with FCR challenging; and 71% were interested in further training in how to manage FCR [22]. Despite evidence of the relationship of FCR with psychological morbidity and impaired quality of life, as well as a recognized need from health professionals, there is a lack of evidence-based treatments for FCR.

Additionally, a growing body of evidence suggests there may also be significant cost savings for the health system if FCR is effectively treated, because people with high FCR use more services or may avoid appropriate tests to identify recurrence in a timely fashion. The former group increase health care use by more frequently presenting for checks, whereas the latter group may present later with a recurrence and therefore require more invasive treatments than might otherwise have been required. Cancer patients with high FCR are: less satisfied with their care [23], more likely to refuse discharge from a cancer centre for follow-up [24,25], seek readmission to a specialised cancer centre [24] and use complementary and alternative medicines [9,26-28]. Higher FCR in young women previously diagnosed with early breast cancer was significantly associated with less frequent than recommended use of mammograms and ultrasounds and more frequent visits to general practioners [9]. Despite these findings, to date, a systematic analysis of the cost-implications of treating FCR has not been conducted.

### Previous interventions

To date few studies have reported an intervention specifically designed to reduce FCR. The first to appear in the literature was a small pilot study of an intervention (6 face-to-face individual sessions with a specialist nurse) [29] based on the self-regulation / common sense model of illness [30,31]. To date no outcome results from this intervention have been published.

Three studies have reported interventions for concepts related but not identical to FCR: fear of cancer *progression*[32-34]. The first evaluated an uncertainty management intervention and the second an intervention to reduce fear of cancer progression (including patients with metastatic disease). However, neither had a clearly articulated theoretical model of these concepts or the interventions used to address them. Moreover, because FCR was not specifically targeted, it was not measured in either study, limiting their utility in this context. Both studies demonstrated an immediate impact of the intervention, but not long-term efficacy. Another non-randomised study involving patients with mixed cancers and disease status has reported data on the efficacy of two interventions to reduce fear of disease progression. Herschbach et al. [34] assessed the impact of cognitive behaviour group therapy and a supportive-expressive group therapy compared to usual care. Patients were assessed prior to the intervention, at treatment completion, and at 3 and 12 months follow-up. Both intervention groups showed a significant reduction in FCR over time compared to the control group. Patients with metastases and cancer recurrence gained the most from the interventions, suggesting that the constructs targeted may have different meanings for disease-free individuals. Subsequent analyses showed that patients with higher education were more likely to have a reliable reduction in FCR [35].

Two further studies have evaluated group therapies designed to improve generic emotional outcomes for cancer survivors, where improvements in FCR were reported as a secondary outcome. The first, [36] involving 84 breast cancer survivors found significant reductions in FCR immediately following a 6-session Mindfulness-Based Stress Reduction group [37], but no long-term data have been published. The second [38] reported significant reductions in FCR immediately following a 12-week Emotion Regulation Group for breast cancer patients which included training in guided imagery, meditation, emotional expression, and exercises promoting control beliefs and benefit-finding. However, improvements in FCR were not sustained at 6 and 12 months post intervention.

In summary, very few studies have evaluated interventions designed specifically to reduce FCR in patients with mixed, early stage cancers, indicating a need for further large, randomised controlled trials of interventions to assist survivors in better managing their FCR.

### Aims

The aims of this study are to evaluate in a randomised controlled trial (RCT), the efficacy and cost efficacy of a therapist-delivered intervention (Conquer Fear) in reducing the impact of FCR in disease-free breast and colorectal cancer survivors with clinical levels of FCR, compared to a relaxation training control intervention. Secondary aims are to evaluate patient satisfaction with the program and the relative cost-effectiveness of Conquer Fear and the relaxation interventions. Specific hypotheses are that:

1. Participants in the Conquer Fear intervention will have lower scores on the Fear of Cancer Recurrence Inventory of at least 14.5 (0.5 standard deviation) as compared to the attention control group (who receive relaxation therapy) at follow-up;

2. Participants in Conquer Fear will have less cancer specific distress, less anxiety and depression, better quality of life, fewer unmet needs, and less maladaptive metacognitions when compared to the control group;

3. Patients receiving Conquer Fear will find the therapy program useful for treating their FCR and be satisfied with the program; and

4. The Conquer Fear intervention will be cost-effective compared to the relaxation comparator intervention.

## Methods/design

This multi-site, parallel randomised controlled trial is being led by the Psycho-Oncology Co-operative Research Group based at the University of Sydney, Australia during 2012–2014. This project was co-funded by *beyondblue,* National Breast Cancer Foundation and Cancer Australia, and registered with the Australian New Zealand Clinical Trials Registry with registration number ACTRN12612000404820. Ethics approval has been obtained from the Cancer Institute NSW Clinical Research Ethics Committee.

### Participants

#### Therapists

Twenty therapists from 15 sites have been recruited to deliver the study interventions via three Australian health professional networks: the Psycho-Oncology Co-operative Research Group (PoCoG), the Australian Psycho-Oncology Group and the Psychologists in Oncology Network. Therapists are eligible to participate in the study if they are registered psychologists, social workers or psychiatrists with a minimum of 5 years professional experience, and 2 years experience in oncology settings. Participating therapists are required to attend a one-day training course involving review of both intervention manuals and practice of the intervention techniques with feedback from senior psychologists/psychiatrists. The training course is based on a previous workshop for therapists who took part in the pilot study of the Conquer Fear intervention [22].

#### Patients

Patients are eligible to participate if they:

•have a confirmed past diagnosis of Stage 0–2 breast cancer or Dukes stage A-C colorectal cancer. These diagnostic groups were selected because they are prevalent; known to be affected by FCR [10,39,40]; have clearly defined staging to allow selection of patients with a good prognosis and in the case of colorectal cancer, include a mix of genders;

•are being treated with curative intent;

•have completed all hospital-based adjuvant treatments at least 2 months and not more than 5 years prior to study entry;

•are disease free;

•have a score in the clinical range (13 or more) on the FCRI Severity Subscale possible range:0–36);

•are able to read and write English;

•are able to give informed consent.

Patients are ineligible if they:

•currently have severe major depression;

•currently are receiving psychological treatment from a therapist not involved in the study;

•currently have active psychotic illness or other psychiatric/cognitive condition.

Patients are recruited from Australian hospitals/cancer centres at which participating therapists are employed. The recruitment method varies between sites due to site-specific feasibility constraints. One recruitment method is for participating therapists to approach oncologists at their site to ask them to identify potentially eligible participants. Invitation letters printed on the sites’ letterhead and signed by the treating oncologist are sent to a random selection of potentially eligible patients in small batches (n = 20) to avoid recruitment of more participants than can be managed by the therapist. A copy of the Severity Subscale of the Fear of Cancer Recurrence Inventory (FCRI) and a contact details form are included with the invitation letter for interested patients to send to the research team. A single reminder phone call is made to all patients invited to take part in the study if they do not respond to the invitation letter after two weeks. Invitation letters ask patients to: consider participation in the study; allow contact by the researchers; complete the Severity Subscale of the FCRI to determine their eligibility for study enrolment.

Alternatively, a study advertisement/flyer is displayed in clinic waiting rooms, or given to patients who express concern over their cancer coming back, by cancer nurse coordinators, therapists (i.e. psychologists or psychiatrists) or treating oncologists. The advertisement advises patients interested in taking part in the study to contact the study coordinators at the University of Sydney. These patients are then sent a copy of the Severity Subscale of the FCRI along with a reply-paid envelope to return to the research team.

Following receipt of the Severity Subscale of the FCRI the study team contacts the patients to confirm whether they are eligible, explains the study in further detail, answers any patient questions, obtains verbal consent, and mails the information sheet and consent form along with the baseline assessment to the patient.

#### Randomisation

Eligible patients who have given informed consent are randomly assigned to receive either the Conquer Fear or relaxation training intervention. The random allocation sequence is generated by a biostatistician blinded to the identity of participants and therapists, using computer-generated random numbers. Randomisation is stratified by therapist, with patients allocated in random blocks of 2 and 4 to enhance balance and reduce the likelihood of researchers or therapists guessing sequence generation. Each time an eligible patient gives informed consent to participate in the study a sequentially numbered envelope containing the patients’ group allocation will be opened by a research co-ordinator who will then forward this information along with the patients contact details to the relevant participating therapist. The biostatistician is blinded to group allocation since the randomization list contains only A versus B and only the research co-ordinators know to which group these refer. The participants are also blinded in that they are not aware which is the primary intervention being evaluated.

### Interventions

#### The theoretical framework of conquer fear

The Conquer Fear intervention is a theory-based intervention developed by a PoCoG-led group including international experts on FCR, psycho-oncology therapists and researchers, medical oncologists, an exercise physiologist, a breast physician and several consumers. The intervention is based on Meta-cognitive Therapy (MCT) [41], the treatment associated with the Self-Regulation of Executive Function (S-REF) Model of emotional disorders [42], together with components of Acceptance and Commitment Therapy [43] to address some of the existential issues related to experiencing a cancer diagnosis. Regarding the latter, it is well documented that cancer survivors commonly face a crisis of life direction: struggling with what life means to them now, what they want to value and see as important and how to lead a life that matters to them and those they love.

Importantly, the S-REF model of emotional disorders provides a mechanism by which high levels of FCR develop and are maintained. It posits that a cognitive appraisal system consisting of self-beliefs, knowledge and beliefs about the benefit and uncontrollability of worry, together with an autonomic appraisal system that evaluates the perceived level of threat associated with physical stimuli, are associated with FCR. Given cancer survivors face a real risk of cancer recurrence, rather than directly challenging the fears associated with a possible recurrence, MCT is well suited to cancer survivors as it works at the metacognitive level teaching clients more effective ways to respond to the presence of fears associated with a potential cancer recurrence. More detailed information regarding the novel theoretical formulation of FCR created by the research team will be published elsewhere.

The key goals of this manualised intervention are to:

•teach strategies for controlling worry and excessive threat monitoring;

•modify underlying unhelpful beliefs about worry;

•develop appropriate monitoring and screening behaviours;

•encourage acceptance of the uncertainty brought about by a cancer diagnosis;

•clarify values and encourage engagement in values-based goal setting.

The intervention is delivered in 5 x 60–90 minute, individual face-to-face sessions by a trained therapist over a period of 10 weeks (sessions are weekly/fortnightly). Each session is accompanied by home-based practice of skills learned in session and home reading to consolidate skill acquisition. See Table 1.

**Table 1:** Detailed content for the conquer fear intervention

**Session**	**Content**	**Home based practice**
1	• FCR-specific assessment;	• Examine values identified in session and devise relevant goals (e.g. if identified value is “being physically fit” devise realistic ways to achieve this and identify barriers to achieving goal);
	• Model on which treatment is based is explained;	
	• Discussion of existential changes brought about by cancer;	
	• Values clarification exercise	
		• Reflect on past experiences and how these have shaped response to cancer.
2	• Discuss impact of potential vulnerability factors (e.g., past traumatic events) on FCR;	• Practice ATT on a daily basis throughout the remainder of the intervention and document in diary.
	• Discuss rationale and practice of the Attention Training Technique (ATT), a technique designed to help patients reduce their tendency to ruminate and shift their attention more flexibly when thoughts about recurrence occur.	
3	• Introduce the practice of Detached Mindfulness, designed to enhance meta-awareness of cognition and the ability to become an objective observer of the content of thoughts without the need for evaluation or reaction.	• Continue daily practice of ATT;
		• Practice application of detached mindfulness on response to thoughts which trigger FCR.
4	• Provide information about possible symptoms of recurrence of breast or colorectal cancer;	• Continue daily practice of ATT;
	• Provide guidelines to help clients distinguish those from benign physical complaints;	• Practice detached mindfulness in response to emerging thoughts about FC;
	• Reassess self-examination practices and medical surveillance;	
	• Identify avoidant or excessive behaviours;	• Devise an appropriate plan based on best available evidence about how to respond to new symptoms;
	• Develop a behavioural contract to help clients to engage in recommended levels of self-examination and follow-up tests (if needed);	
	• Discuss beliefs that underpin FCR (eg. beliefs about the benefits of FCR, or beliefs about physical harm caused by FCR);	• Practice worry postponement.
	• Test the validity of these beliefs through Socratic dialogue.	
	• Introduce worry postponement as a technique for responding to residual worries	
5	• Review goal setting task;	
	• Consolidate skills learned throughout the program;	
	• Develop relapse prevention plan.	

#### Relaxation training

Participants randomised to the control treatment receive a 5-session relaxation training program over a period of 10 weeks, as detailed in Table 2. Participants receive training in progressive and passive muscle relaxation, meditative relaxation, visualisation and “quick relaxation” techniques, with regular home-based practice and a CD for practice outside sessions. The aim of the control condition is to provide an equivalent amount of therapist contact to Conquer Fear, to control for non-specific therapeutic factors (e.g. therapeutic alliance and support), in a treatment package with face validity so that patients are not aware they are in the control condition. Relaxation therapy may have some beneficial effects itself.

**Table 2:** Detailed content for the relaxation training intervention

**Session**	**Content**	**Home based practice**
1	• Assess FCR;	Practice active PMR daily for 25 minutes
	• Explain concept of stress, how it can be adaptive when it becomes a problem and how it can be managed through relaxation;	
	• Introduce different stress management techniques;	
	• Practice active progressive muscle relation (PMR);	
2	• Review active PMR and its impact on stress levels;	Practice passive PMR daily for 25 minutes
	• Identify and resolve issues of practicing active PMR;	
	• Introduce passive PMR;	
	• Practice passive PMR;	
3	• Review passive PMR and its impact on stress levels;	Incorporate meditative relaxation into passive PMR practice for 25 minutes daily
	• Identify and resolve issues of practicing passive PMR;	
	• Introduce meditative relaxation;	
	• Practice meditative relaxation.	
4	• Review meditative relaxation and its impact on stress levels;	Incorporate visualisation into passive PMR practice for 25 minutes daily
	• Identify and resolve issues of practicing meditative relaxation;	
	• Introduce visualisation as a way of shortcutting the stress response;	
	• Practice visualisation.	
5	• Review visualisation and its impact on stress levels;	
	• Identify and resolve issues of practicing visualisation;	
	• Introduce quick relaxation techniques;	
	• Practice quick relaxation;	
	• Review skills learnt and progress made;	
	• Develop plan for future practice of relaxation techniques.	

#### Intervention fidelity checks

Fidelity to treatment protocols in both arms will be ensured by: a) having therapists submit a completed checklist following each session; b) regular review of submitted session checklists; c) therapist participation in monthly group supervision; and d) recording of therapy sessions. A random selection of 10% of recordings will be assessed by the research team using a modified version of the revised cognitive therapy scale (CTS-R; [44]). The scale was designed to assess therapist competency in cognitive therapy, and has good psychometric properties [44]. The scale assesses skills specific to cognitive therapy, general therapeutic skills including therapeutic alliance and has been modified to include competency in skills necessary for the FCR intervention that are not otherwise covered by the CTS-R. Feedback will be given where non-fidelity is identified, as recommended by the validated ‘Method of Assessing Treatment Delivery’ [45].

#### Pilot study

A pilot study of the intervention was carried out between October 2010 and December 2011 [22]. Results are presented in detail elsewhere [22]. In brief, ten psychologists / psychiatrists took part in a 1-day training session held in October 2010. In post-training questionnaires, all therapists had increased knowledge about and confidence in managing FCR. At the close of the pilot 4 therapists had recruited 8 participants (2 each). Recruitment was 83% and retention rates 100%. Both patients and therapists were highly satisfied with the intervention. Patients reported finding the intervention very helpful and had a significant and clinically meaningful reduction in FCR as measured by the FCRI.

### Outcomes

All participant outcomes are assessed using either an online or paper and pencil self-report questionnaire containing the measures listed below. Participants are posted or e-mailed, as preferred, the questionnaires, to be completed and returned in a reply-paid envelope or online. Questionnaires are completed upon enrollment in the study prior to randomisation, immediately after treatment completion, 3 and 6 months after treatment completion. The researchers follow up participants who do not return their questionnaire within two weeks, with up to three phone calls at different times of the day according to a pro forma. If phone contact cannot be made, email and text reminders are sent where possible. Finally, a replacement questionnaire is sent 3 weeks after the non-returned questionnaire.

#### Primary outcome

Fear of Cancer Recurrence is assessed using the 42-item FCRI [39], the most comprehensive multi-dimensional scale of FCR available, and suitable for patients with mixed cancer diagnoses. It has been found to be internally consistent (Chronbach’s α=0.75 to 0.91 across subscales) and stable over a two-week interval (ρ = 0.58 to 0.83 across subscales), and has a robust factor structure. The FCRI has convergent validity with other standardised measures of FCR (ρ = 0.66 to 0.77) and discriminant validity with QOL amongst a large sample (n = 600) of Canadian cancer patients with mixed tumours [39]. Respondents rate the degree to which symptoms or issues affect them on a Likert scale ranging from 0 (‘not at all’ or ‘never’) to 4 (‘a great deal’ or ‘all the time’). Higher scores indicate higher FCR.

#### Secondary outcomes

•Cancer-specific distress is assessed with the 22 item Impact of Event Scale [46] with two subscales: intrusion and avoidance.

•General distress is assessed with the Depression, Anxiety, Stress Scale, 21 item short form [47,48].

•Quality of life is assessed with the AQoL8D, a 35 item health-related QOL instrument that includes domains of mental health, coping, self-worth, happiness, relationships, along with the other subscales assessing functional aspects of QOL; independent living, pain, and senses. The AQoL8D [49,50] also allows the calculation of quality-adjusted-life years (QALYs) through the health state utility scoring algorithm.

•Unmet needs are assessed using the 8 item Information sub-scale of the Survivors Unmet Needs Survey, which includes needs relating to finding information, knowing which information to trust and worrying about cancer recurrence and spread [51].

•Treatment satisfaction is evaluated using a single question assessing overall satisfaction with the therapy.

#### Exploratory outcomes

•Metacognitions (the cognitive processes, strategies, and knowledge that are involved in the regulation and appraisal of thinking itself.’[52]) are measured with the Metacognitions Questionnaire-30 [52,53], a 30 item questionnaire with 5 subscales measuring: cognitive confidence; positive beliefs about worry; cognitive self-consciousness; uncontrollability and danger; and need to control thoughts.

•Fear of cancer recurrence will also be measured using the Concerns About Recurrence Questionnaire (CAR-Q), a 5 item purpose designed questionnaire developed by BT and PB, to act as a brief screen of FCR. This is to test the psychometric properties of the CAR-Q.

#### Potential covariates

Clinical and demographic information will include age, time since diagnosis, marital status, education, parity, stage of disease, treatment received (both medical and psychological) and current treatment (e.g. anti-depressants (if any)). In addition individual level of perceived risk of cancer recurrence will be assessed as this may influence firstly level of FCR prior to treatment and also treatment efficacy. Other potential covariates related to treatment are therapeutic alliance and treatment expectancy and credibility. Therapeutic alliance, which has consistently been shown to be associated with treatment outcome [54], will be assessed using the 12-item Working Alliance Inventory short-form revised (WAI-SR) [55]. The WAI-SR assesses the agreement on goal, the agreement on tasks that achieve the goal, and bond between patient and therapist. Treatment expectancy, which has been shown to predict treatment outcomes in a variety of contexts [56], will be measured using the 6-item Credibility/Expectancy Questionnaire [57].

#### Therapist measures

Training evaluation is completed by participating therapists immediately after training completion. The evaluation survey includes items surveying: basic demographic features, professional qualifications and experience, confidence in dealing with FCR pre- and post-training, and satisfaction with the intervention training content and delivery using purpose designed Likert scales. This survey was used in our previous pilot study [22].

#### Economic measures and analysis

Economic evaluation will be a cost-consequences analysis conducted from both the broad societal perspective and the narrower perspective of the health care sector. The economic evaluation will compare any incremental costs of the intervention (costs accrued in the intervention arm compared to costs accrued in the control arm) to the full list of incremental outcomes (including QALYs in a cost-utility analysis). The intervention costs in the evaluation will include only the costs of intervention delivery, (excluding development or research costs), to estimate the resource use required if the intervention was rolled-out into practice across Australia. The research team and provider records will be used to determine the costs of the intervention (screening costs, cost of intervention delivery and intervention materials).

Other costs associated with each arm of the trial will be estimated using a resource use diary. The diary will be used to document and measure health behaviours and service use - such as hospitalisation, other allied health consultations, use of alternative therapies and self-monitoring behaviours. The diary will also document days out-of-role (including paid and unpaid work) travel costs and carer costs. The diary will be based on existing resource use questionnaires used in national databases and will cover both costs paid for by the individual as well as those paid for by third parties (such as an insurance company). Measured resource use will be valued using existing unit costs from sources such as the Australian Refined Diagnosis Related Groups (AR-DRG). AR-DRG for costs of hospitalisation and Australian Bureau of Statistics estimates of Australian earnings for productivity effects. Based on previous research, questionnaires have been found to be reliable estimates of resource use [58].

Uncertainty in the cost and outcome data will be further evaluated through nonparametric bootstrap analysis. The sensitivity of economic evaluation results to the methods of evaluation will be tested through sensitivity analyses, whereby key evaluation parameters (such as unit costs), are varied to assess the impact on the study conclusions.

#### Sample size

This is a longitudinal randomised controlled trial, with one baseline measure and 3 post-intervention measures. The primary outcome is the Fear of Cancer Recurrence Inventory, a 42 item scale with a possible range of 168.

We based our sample size calculation on 90% power, a two-sided α = 0.05 t-test, dropout of 30%, a difference between groups of 14.5, and a standard deviation of 29 [39] (a standardised effect size of 0.5). The sample size calculations should account for the clustering of outcomes by therapist, and although there is no published intra-cluster correlation (ICC) for the FCRI, we took guidance from two large reviews on ICCs in similar contexts [59,60] and used an ICC of 0.01. Assuming a total of 20 therapists, and following Donner and Klar, [60,61] the required sample size is 13 patients per therapist, for a total of 260 patients. If dropout is lower, for example, 20% then this sample size allows for a larger intra-cluster correlation or a small detectable difference. This sample size also gives 90% power to detect a difference of 0.5SD between the groups in each of the secondary outcomes. The value includes attrition of 30%, however, we expect attrition to be lower, since patients with FCR report a high level of need for help, and we experienced low attrition when piloting the intervention.

#### Statistical analysis

Analyses will be by intention-to-treat. Linear mixed models will be used to account for the hierarchical, non-independent nature of the data: repeated measures on patients nested within therapist. A saturated time x treatment model with unstructured covariance structure will be used to guard against misspecification of the model. Random effects of patient and therapist will be used. The primary analysis will be the comparison at the each of the 3 post-baseline (post-treatment) time points of the two arms using a contrast within this mixed model. Overall pattern of change will be assessed by testing the treatment by time interaction. Adjusted analysis, including using baseline FCRI, sex, age, time since diagnosis, cancer type, perceived risk, treatment expectancy and therapeutic alliance will also be investigated. Variables will be included in the model if they have a Kendall’s Tau correlation higher than 0.1 with FCRI. Mixed models yield unbiased estimates for data which are missing completely at random and at random [62], however, if the amount of missing data for the primary outcome is greater than 20%, sensitivity analyses will be performed by multiply imputing the missing data, and adding and subtracting clinically plausible amounts from the imputed values. These new datasets will be re-analysed, to see how conclusions change.

Secondary outcomes (QoL, distress, unmet information needs) will be analysed in a similar way to the primary outcome. Treatment satisfaction and therapist training evaluation will be described with descriptive statistics. To see if colorectal and breast cancer patients responded differently to the treatment, we will test the interaction cancer type x treatment x time. Predictors of FCR, distress and QoL at baseline will be explored using multiple linear regression. The relationship between Metacognition and FCR will examined using 1) regression to see if there is a relationship at baseline in the entire sample and then 2) using mediation techniques following the methods of Baron and Kenny [63] in the treatment group only, immediately post baseline (time 1). The CAR-Q will be compared with the FCRI using psychometric techniques.

## Discussion

### Theoretical significance

This RCT advances the field by representing the first controlled trial of a novel, evidence-based and theoretically-based model of psychological support for cancer survivors with high FCR. Further, the inclusion of a health economic evaluation will strengthen the study further. Given the dearth of intervention research anchored in theoretical frameworks, this trial is an important next step in filling the gap in knowledge about how best to support this large and growing group. Extensive preliminary research and pilot work has demonstrated the likely success of this model.

### Clinical significance

The Conquer Fear intervention aligns with emerging priorities in survivorship care in cancer which identify promotion of quality of life and maximizing well-being and optimal participation in social and occupational roles as central to care, rather than the traditional surveillance-based approach to follow-up which focuses on identification of new disease or late complications of cancer treatment. It is also in line with consumer priorities for survivorship research [64]. This intervention has the potential to reduce FCR and improve the QoL of patients, and reduce health care costs. The eventual goal of this study is to distribute an evidence-based, manualised, psychological intervention for FCR. Implementation of the Conquer Fear intervention will therefore help to eradicate barriers to appropriate psycho-social care for survivors, and has far-reaching service and policy implications in terms of implementing ‘best practice’ recommendations for the ever-increasing numbers of cancer survivors.

## Abbreviations

FCR: Fear of cancer recurrence; RCT: Randomised controlled trial; FCRI: Fear of cancer recurrence inventory; CAR-Q: Concerns About Recurrence Questionnaire; S-REF: Self-Regulation of Executive Function; ATT: Attentional training technique; PMR: Progressive muscle relation; CTS_R: Revised cognitive therapy scale; QALY: Quality-adjusted-life year; WAI-SR: Working Alliance Inventory short-form revised; AR-DRG: Australian Refined Diagnosis Related Groups; ICC: Intra-cluster correlation

## Competing interests

The authors declare that they have no competing interests.

## Authors’ contributions

PB, BT, JT, JG, JB, AG, LS, MB & CM were responsible for the conception and initial study design. ABS, JEF and SS were responsible for refining the study design and will be responsible for co-ordinating the acquisition of study data. MB, and SS will be responsible for analysis of study data. All authors were involved in drafting the manuscript and have read and approved the final manuscript.

## Pre-publication history

The pre-publication history for this paper can be accessed here:

http://www.biomedcentral.com/1471-2407/13/201/prepub
